# Text Simplification Using Consumer Health Vocabulary to Generate Patient-Centered Radiology Reporting: Translation and Evaluation

**DOI:** 10.2196/jmir.8536

**Published:** 2017-12-18

**Authors:** Basel Qenam, Tae Youn Kim, Mark J Carroll, Michael Hogarth

**Affiliations:** ^1^ Department of Radiological Sciences College of Applied Medical Sciences King Saud University Riyadh Saudi Arabia; ^2^ Health Informatics program School of Medicine University of California, Davis Sacramento, CA United States; ^3^ Betty Irene Moore School of Nursing University of California, Davis Sacramento, CA United States; ^4^ Division of Health Informatics Department of Public Health Sciences University of California, Davis Sacramento, CA United States; ^5^ Division of Pathology Informatics Department of Pathology and Laboratory Medicine University of California, Davis Sacramento, CA United States; ^6^ UC San Diego Health University of California San Diego, CA United States

**Keywords:** consumer health information, vocabulary, radiology, electronic health records, natural language processing

## Abstract

**Background:**

Radiology reporting is a clinically oriented form of documentation that reflects critical information for patients about their health care processes. Realizing its importance, many medical institutions have started providing radiology reports in patient portals. The gain, however, can be limited because of medical language barriers, which require a way for customizing these reports for patients. The open-access, collaborative consumer health vocabulary (CHV) is a terminology system created for such purposes and can be the basis of lexical simplification processes for clinical notes.

**Objective:**

The aim of this study was to examine the comprehensibility and suitability of CHV in simplifying radiology reports for consumers. This was done by characterizing the content coverage and the lexical similarity between the terms in the reports and the CHV-preferred terms.

**Methods:**

The overall procedure was divided into the following two main stages: (1) translation and (2) evaluation. The translation process involved using MetaMap to link terms in the reports to CHV concepts. This is followed by replacing the terms with CHV-preferred terms using the concept names and sources table (MRCONSO) in the Unified Medical Language System (UMLS) Metathesaurus. In the second stage, medical terms in the reports and general terms that are used to describe medical phenomena were selected and evaluated by comparing the words in the original reports with the translated ones. The evaluation includes measuring the content coverage, investigating lexical similarity, and finding trends in missing concepts.

**Results:**

Of the 792 terms selected from the radiology reports, 695 of them could be mapped directly to CHV concepts, indicating a content coverage of 88.5%. A total of 51 of the concepts (53%, 51/97) that could not be mapped are names of human anatomical structures and regions, followed by 28 anatomical descriptions and pathological variations (29%, 28/97). In addition, 12 radiology techniques and projections represented 12% of the unmapped concepts, whereas the remaining six concepts (6%, 12/97) were physiological descriptions. The rate of lexical similarity between the CHV-preferred terms and the terms in the radiology reports was approximately 72.6%.

**Conclusions:**

The CHV covered a high percentage of concepts found in the radiology reports, but unmapped concepts are associated with areas that are commonly found in radiology reporting. CHV terms also showed a high percentage of lexical similarity with terms in the reports, which contain a myriad of medical jargon. This suggests that many CHV terms might not be suitable for lay consumers who would not be facile with radiology-specific vocabulary. Therefore, further patient-centered content changes are needed of the CHV to increase its usefulness and facilitate its integration into consumer-oriented applications.

## Introduction

### Engaging Patients in Health Care Processes

The modern view of medicine endorses engaging patients in their health care [1]. These efforts have been facilitated by the accelerating adoption of health information technology in the United States [2] after the Health Information Technology for Economic and Clinical Health (HITECH) Act [3]. In 2008, the American Medical Informatics Association’s Consumer Health Informatics Working Group identified the need for consumer-oriented tools to improve consumers’ understanding of health information [4]. A Web-based patient portal, for example, is an important communication form between the patient and the provider that can increase the transparency of health care processes [5]. A multicentral experimental study conducted in 2012 suggests that a majority of people who accessed their clinical notes through the Web sensed more control over their health care processes and showed an improved adherence in taking medications [6]. Therefore, a number of large health care delivery organizations in the United States have adopted initiatives such as Open Notes, which grant patients a secure access to their medical records though Web portals [7].

### Radiology Reports

Radiology reports are one of the documents that have become unprecedentedly open to patients in multiple medical institutions nationwide [5,8]. However, these reports use clinical and radiology terms that are unfamiliar to the lay public, which creates an opportunity for significant misinterpretation by patients.

In the traditional health care process, radiology reports are where radiologists professionally express their knowledge and expertise to other physicians. The reports serve the purpose of describing diagnostic images to look for abnormalities, leading radiology reports to include a myriad of anatomical structures and pathological concepts. This makes it difficult to interpret such texts for someone without knowledge in the field.

Generally, clinical documentation requires average reading skills higher than those of adults in the United States [9]. Understanding many forms of clinical notes requires formal training in medical terminology. The level of complexity, nonetheless, differs from one type of documentation to another. In comparison with visit summaries, for instance, radiology reports are considered one of the most difficult clinical documentation forms for the lay public to understand [10]. Therefore, providing such clinically oriented reports to patients through Web portals without considering their medical literacy level can be irrelevant and problematic. Reports should continue, nevertheless, to provide the clinical value and accuracy, and adhere to the needed time efficiency of the clinical workflow. Although the heterogeneity in the audience may cause contradictions in the requirements of a radiology report, it is costly and time-consuming to manually create an additional consumer-friendly version of the report. As a result, an automated maneuver to create a simplified version of the radiology report would be desirable.

### Lexical Simplification

Text simplification is a division of natural language processing (NLP) that can refer to several syntactic, semantic, and lexical methods with the goal of simplifying text [11,12]. Early work in the field of text simplification focused on simplifying Web and newspaper articles for language learners and people with disorders that negatively affect reading abilities [13]. The focus of text simplification is to produce a more readable and understandable text for the reader, without considering if it is shorter or longer. This distinguishes it from text summarization, where the major goal is text shortening. Lexical simplification, for example, is one realm of text simplification that focuses on replacing terms in a context with simpler synonyms [14]. This can be useful when a main reason behind text difficulty is that it includes terms unfamiliar to the readers, which is the case in radiology reports [10].

One important part of lexical simplification is to identify difficult terms and represent those with what are considered simpler terms. Such determination, nevertheless, tends to be subjective and ambiguous, which makes it hard to conduct by a computer. Usually, automated methods used to assign difficulty levels for words include measuring words length, the number of syllables, and usage frequency [15,16]. In different realms of medicine, however, there are ontologies that can predefine medical terms, which might require simplification for the public. For example, RxNorm includes medications’ names for every drug in the US market [17], and the Logical Observation Identifiers Names and Codes (LOINC) is a collection of terms used in laboratory observations [18]. Furthermore, to harmonize these efforts, the National Library of Medicine built the Unified Medical Language System (UMLS), integrating similar terms from different ontologies into unified entities, each represented by a concept unique identifier (CUI). These data are constructed in relational tables that form the UMLS Metathesaurus [19]. MRCONSO is one of the tables in the database, and it includes preferred terms and their synonyms linked to CUIs and other identifiers from the original sources of the terms (ie, LOINC).

### The Open-access, Collaborative Consumer Health Vocabulary (CHV)

The UMLS Metathesaurus also includes the open-access, collaborative consumer health vocabulary (CHV) [20] developed by the Biomedical Informatics Department at the University of Utah as an open-source set of biomedical terms that are suitable for laypersons. Research that uses the term *consumer health vocabulary* started in 2003 [21], with the purpose of helping the lay public to understand health information [20]. CHV is a collection of terms found to best represent the medical concepts for consumers; they are chosen because they are more comprehensible by patients when compared with their synonyms [22].

The CHV has been implemented in the literature as the back end of medical lexical simplification techniques for consumers. This has been, especially, possible because the CHV concepts are linked to the medical concepts in the UMLS. In 2007, Zeng-Treitler et al tested the use of CHV as the basis of a translator prototype that attempted to simplify the content of electronic medical records and biomedical literature [23]. Zeng-Treitler’s model changes terms to CHV-preferred terms when available and, if not, provides “explanations” based on the UMLS hierarchal and semantic relations [23]. The correct translations reported in the latter model were considered promising, yet the percentage of incorrect translations was 8.2%. Most of the inaccuracy, as explained, was because of incorrect hierarchical relations in the UMLS such as “tobacco abuse” as “a type of psychiatric problem.” The semantic relationships among concepts included in the UMLS Metathesaurus are suboptimal because of the UMLS content being derived from external terminological systems that use a variety of heuristics for conceptual relationships.

Hypothetically, the CHV can help in creating a more consumer-oriented version of radiology reports. Yet, there is a lack of evidence on whether CHV is an appropriate source of lay terms that can simply replace medical terms used in the field of radiology, which has unique contexts and jargon. Any implementation of CHV should take lexical differences into consideration to make sure CHV is a good fit for the required purpose. Text simplification is by nature more sensitive to errors than other NLP techniques. Incorrect translations can make clinical reports more difficult to comprehend and might provide incorrect information to patients, such as defining a “cyst” as “a type of tumor” [23]. Higher comprehension of radiology concepts would lead to relying on less hierarchal relations, which was shown to introduce more errors.

We believe that automated simplification of radiology reports using lay terms would be beneficial to patients as such simple transformation of medical terms could enhance patients’ understanding of the content. The first step in this endeavor is to determine the relevance and comprehension of CHV as a backbone of a lexical simplification tool for radiology reports. The objectives of this study can be conceived by answering the following set of questions:

- What is the content coverage of CHV for concepts included in radiology reports?
- What is the percentage of lexical similarity between CHV-preferred terms and medical terms used in the radiology reports?
- What are the main observations and obstacles in implementing CHV in a lexical simplification tool for radiology reports?

Although content coverage shows the level of comprehension on covering radiology concepts, lexical similarity can indicate how the CHV distinctively describes concepts in a consumer-oriented manner.

## Methods

### Overview

**Figure 1:**
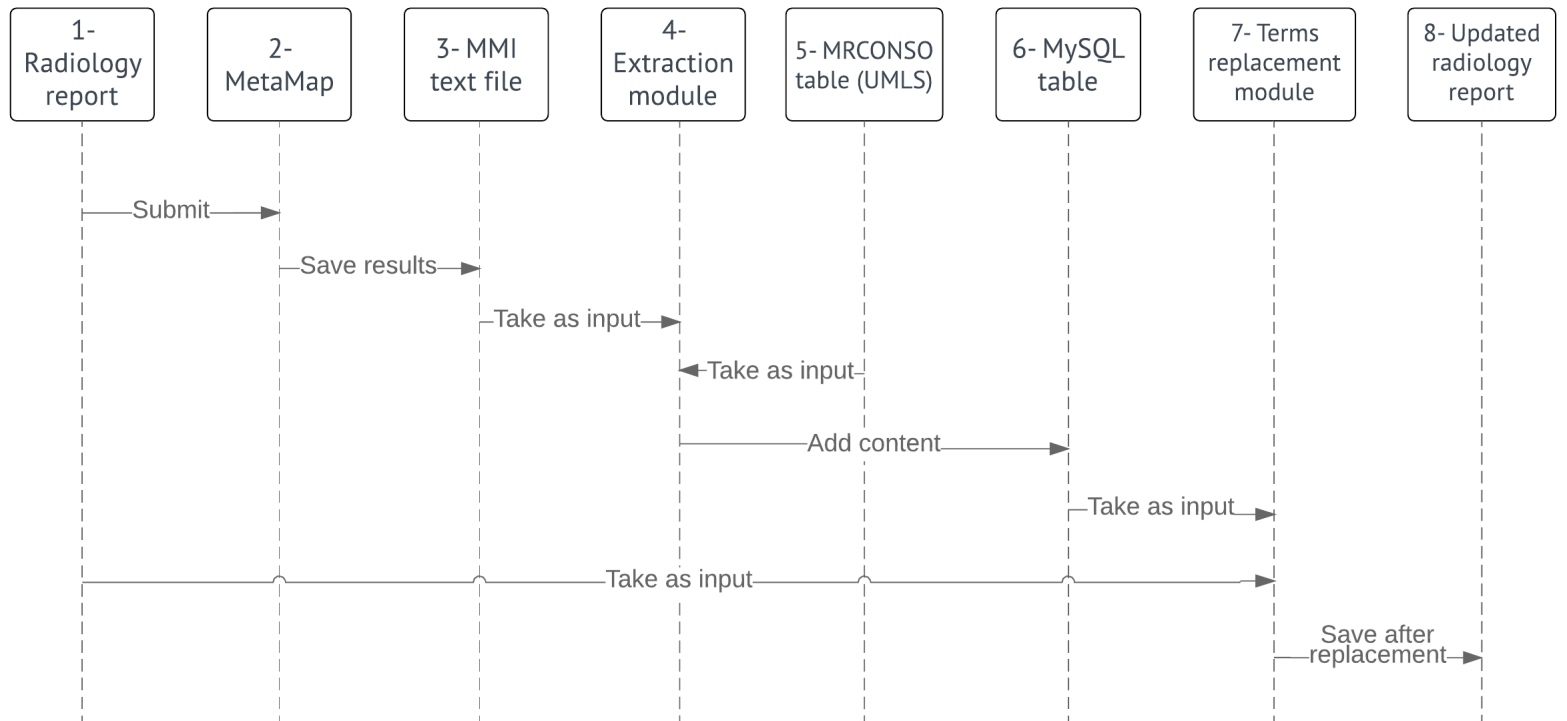
A sequence diagram of the translation process. UMLS: unified medical language system; MMI: MetaMap indexing.

The overall procedure can be divided into the following two main stages: (1) translation and (2) evaluation. The translation process involving the following two components is outlined in Figure 1: mapping medical terms to CHV concepts, which corresponds to the objects 1 to 3 in the diagram, and replacing medical terms in the reports with CHV-preferred terms (objects 4-8 in Figure 1).

### The Translation Process

#### Mapping Terms in Radiology Reports to Their Relevant Concepts in the Consumer Health Vocabulary

A set of 31 radiology reports were parsed and mapped to the CHV concepts using MetaMap 2016 [24], an NLP tool created by the National Library of Medicine based on the UMLS Metathesaurus. The reports were anonymous Web-published samples from multiple institutions in the United States [25-28]. The sample comprised 10 magnetic resonance imaging (MRI) reports, 6 ultrasound reports, 5 computed tomography reports, 5 nuclear medicine reports, and 5 x-ray reports. The anatomical structures covered in these imaging acquisitions included the abdomen, the chest, the neck, the head, the shoulders, and the ankles. In addition, there were organ-specific acquisitions for the brain, the spine, the kidneys, the liver, and testicles. This was besides two physiology-oriented cases, a Doppler carotid scan and a carotid stress test.

In the mapping process, all the semantic types in the UMLS were included [29], and a low MetaMap evaluation score threshold of 500 out of 1000 was set [30]. Both decisions had been made to ensure that the system is as inclusive as possible in linking report terms to CHV concepts. Restricting semantic types reduces mapping errors caused by linking terms to concepts in irreverent categories (ie, geographical area). It can, nevertheless, affect the comprehension of the tool in detecting some applicable concepts if their semantic types were excluded. Similarly, a low evaluation score threshold would spread the net for the purpose of measuring the content coverage. Although this would increase the risk of mapping words or terms to irrelevant concepts, these incidents were excluded through manual investigation.

#### Replacing Every Mapped Term in the Reports With the Consumer Health Vocabulary–Preferred Term of its Representative Concept

In the previous process, the output of MetaMap was created in the form of fielded MetaMap indexing (MMI), a pipe-separated structured text document that could be processed with programming scripts. One program using Python programming language [31] was created to extract the CUIs of the detected concepts and the terms that provoked each CUI. This program can detect up to 4 terms for each CUI and load all extracted pieces of data into a MySQL [32] database. In addition, it adds the CHV-preferred terms to a table by matching the detected CUIs in MRCONSO. Another script replaces the list of terms in the table with their CHV-preferred terms counterparts. To facilitate pinpointing during evaluation, the replaced words were marked with square brackets, such as [CHV Preferred Term], to be recognizable for evaluation.

### The Evaluation Process

After completing the term replacement process, we counted medical terms in the reports, such as anatomical structures and abnormalities. Additionally, we included general terms that have been used to describe clinical phenomena and might need clarification, such as “irregular” to describe the margins of a mass. Words, however, that are considered simple English, for instance “patient” or “right side,” were not counted despite being mapped to CHV concepts. When a medical term was not mapped to a CHV concept, it was investigated in the CHV Web portal to eliminate the chances of false negatives in the coverage because of limitations in the mapping process. Similarly, words that had been mapped to incorrect concepts were excluded if they did not have matching concepts in the CHV after a manual investigation. Each term is counted once unless it was written once in full and used again as an abbreviation.

To measure the content coverage of CHV and lexical similarity of terms between the radiology reports and CHV-preferred terms, included terms were classified in two ways. First, to measure the content coverage, terms that are represented by CHV concepts were compared with terms that did not match any CHV concept, following the equation:

Content coverage = (Covered terms × 100) / (Total terms)

Second, terms mapped to CHV concepts were categorized based on their lexical similarity with the original terms used in radiology reports. Lexical similarity refers to different statistical calculations that are commonly used to measure how close two different languages or two pieces of text are to each other [33]. In this context, it is calculated using the following equation:

Lexical similarity = (Similar terms × 100) / (Covered terms)

A word is considered lexically similar if its CHV-preferred term is the same word or a variant that is based on the same stem. Additionally, phrases are considered similar if the same words are paraphrased differently (Table 1).

Terms are considered lexically different if their CHV-preferred terms include words derived from distinct stems. This includes preferred terms with added words such as replacing the term “ventricles” with “heart ventricles,” as well as any terms that were totally altered, such as replacing “necrosis” with “tissue death.” The importance of lexical similarity is based on the premise that consumer-oriented terms are supposed to be expressed differently in comparison with clinically oriented reports that are characterized by being difficult for consumers to grasp.

**Table 1:** Examples of lexical similarity classification.

Report term	Preferred term	Lexical similarity
Lateral	Lateral	Similar
Thickening	Thickened	Similar
Abdominal wall	Wall of abdomen	Similar
Ventricle	Heart ventricle	Different
Necrosis	Tissue death	Different

## Results

### Content Coverage

**Figure 2:**
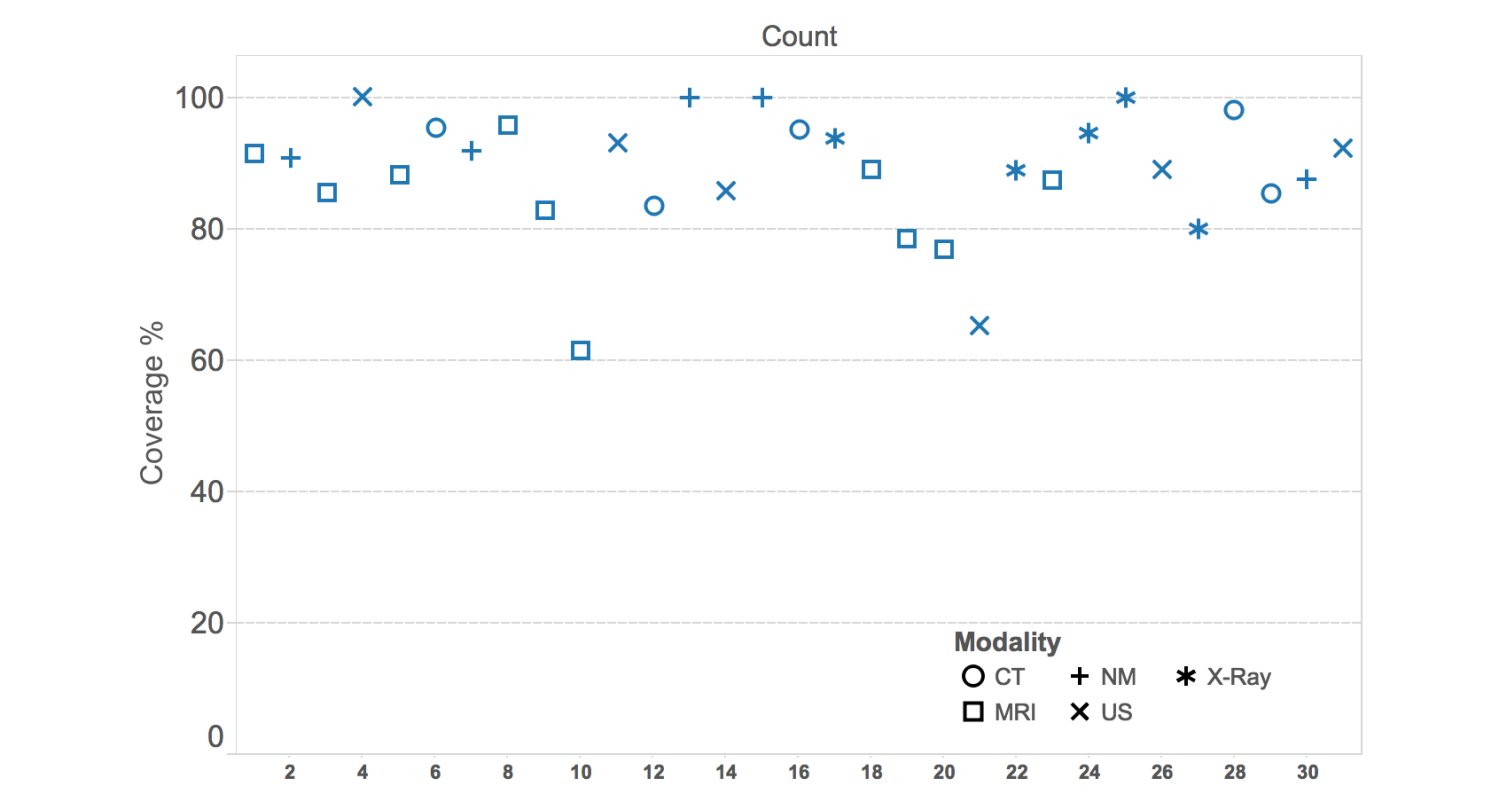
A scatter plot of sample reports percentages of content coverage. CT: computed tomography; MRI: magnetic resonance imaging; NM: nuclear medicine; US: ultrasound.

Out of 792 terms extracted from the sample of 31 reports, 695 terms (88.5%) were covered by CHV concepts (Table 2). Coverage per report ranges from 61% to 100% (Figure 2), with an average of 88.5% and a median of 88.9%.

When categorizing the reports based on imaging modalities (Table 3), content coverage ranges approximately between 84% and 94%, with ultrasound being the lowest and nuclear medicine being the highest. For the results of individual reports’ analyses please see Multimedia Appendix 1.

### Missing Concepts

The analysis shows that 97 terms of the 792 terms (12.2%) were not linked to CHV concepts. To illustrate the coverage gaps found in radiology reports, uncovered terms can be divided into the following four categories:

1. *Human anatomical structures and regions:* 51 terms (53%) are in this category. Examples include “disc space” and “midthoracic.”
2. *Anatomical descriptions:* For example, “heterogeneity” and normal and pathologic variations such as “tracheomegaly” represented 28 of the unmapped terms (29%).
3. *Radiology-related techniques and projections:* For example, “post-contrast” and “anteroposterior” accounted for 12 missing terms (12%).
4. *Physiology-related terms:* Six uncovered terms (6%) are considered in this category. Examples are “metabolically active” and “basic” as used in “basic rhythm of atrial fibrillation.”

**Table 2:** A summary of terms classification, content coverage, and percentage of similar terms in 31 sample reports.

Category of terms	Similar	Different	Missing	Total covered	Total
Sum	505	190	97	695	792
Percentage (%)	63.8	24	12.2	88.5	100

**Table 3:** Sample reports classified per modality, showing the content coverage and lexical similarity for each set, and the average coverage and similarity for the different modalities.

Modality	Reports’ quantity	Similar terms	Different terms	Missing terms	Total terms	Covered terms	Coverage (%)	Similarity (%)
Magnetic resonance imaging	10	229	91	58	378	320	84.66	71.56
Ultrasound	6	52	16	13	81	68	83.95	76.47
Computed tomography	5	90	39	11	140	129	92.14	69.77
Nuclear medicine^a^	5	81	22	7	110	103	93.64	78.64
X-ray^b^	5	53	22	8	83	75	90.36	70.67
Average							88.95	73.42

^a^Includes 1 report of a positron emission tomography-computed tomography procedure.

^b^Includes 1 report of a mammography procedure.

**Table 4:** A summary of terms’ classification based on lexical similarity. This classification only includes terms that are covered by the consumer health vocabulary.

Terms	Similar	Different	Total
Sum	505	190	695
Percentage (%)	72.6	27.3	100

In a linguistic manner, there are areas where the CHV fails to cover concepts more than others. Two main areas are compound words and words with affixes such as “extraaxial” and “paracentral.” Another challenge is abbreviations. In many cases, the original terms were present in CHV, but not their acronyms. An example of this is the phrase “left ventricle,” which is present in CHV, but not the abbreviation “LV.” Figure 2 illustrates 2 reports that are noticeably lower in coverage than others, a lumber MRI report being the lowest, followed by a report of a carotid Doppler ultrasound procedure. Although the MRI case mostly contains uncovered anatomical structures, the Doppler ultrasound missed abbreviations to express names of arteries.

### Lexical Similarity

Looking at the terms covered by CHV concepts, the average lexical similarity between the CHV-preferred terms and the terms in the radiology reports was approximately 72.6% (Table 4).

Approximately 27.3% of the covered terms were replaced with terms or phrases that are lexically different from what was written in the reports. In some cases, words were replaced with more familiar descriptions, such as replacing the word “necrosis” with “tissue death” or replacing “cortex” with the phrase “outer layer of an organ.” Other terms were replaced with phrases that include the original terms but with a brief illustration, such as “heart ventricles” in place of “ventricles.” In addition, few terms were replaced with others that can be less familiar. For example, the CHV-preferred term “effusion” replaced the phrase “free fluid,” which was used in the reports.

### Other Observations

#### Idiosyncrasies With Semantic Types

When only semantic types of interest were chosen, a concept was found for the word “anterior,” but not the word “posterior.” As they have an antonymous relationship, one might expect they are in the same semantic category, yet “anterior” is classified in the UMLS as a “functional concept,” whereas “posterior” is a “spatial concept,” a type that was excluded in this experiment. When semantic types were not specified, there were more mappings to irrelevant concepts. For example, the word “no” was mapped to the concept “nitric oxide,” when the semantic types “Biologically Active Substance” or “Pharmacologic Substance” were not excluded. In addition, there were more mappings to words with the wrong sense, word sense disambiguation errors. For example, the word “ultrasound” was mapped to the concept “therapeutic shock waves,” although the meaning of the word in the report refers to “diagnostic ultrasound.”

#### Syntactic Errors

In some cases, when replacing terms with others, part of speech of the words is altered, such as when the noun “opacity” replaces the adjective “opaque.” Furthermore, articles, “a” and “an” needed an adjustment after replacing a word that begins with a consonant with another word that starts with a vowel.

#### Spelling Errors

The term “homogenous” was written in one report to describe what is usually referred to as “homogeneous.” This has led to mapping the term to a different concept “homozygote,” which is not the meaning in the report. Another case is the term “metastatic,” which is misspelled in one report to be “metastic.” In that case, the term was not mapped to a concept.

#### Representation of Consumer Health Vocabulary in the Unified Medical Language System

In the MRCONSO table, it is possible to find 2 CHV terms marked as preferred term with the same UMLS CUI, although only one is expected. As the mapping results from MetaMap are CUI-oriented, there has been an ambiguity in defining the right CHV-preferred terms in these cases from the MRCONSO table. The situation was different, nonetheless, when searching for a CUI directly in the CHV Web portal. For example, the word “change” has 2 CHV-preferred terms in the MRCONSO table, “change” and “modified,” yet only one preferred term, “change,” was found in the CHV Web portal. This is true for all other concepts in the Web portal, but there has not been a pattern to define that single term in the MRCONSO table.

## Discussion

### Principal Findings

Of the 31 radiology reports examined, the CHV shows an overall high percentage of content coverage (88.5%) for terms found in sample reports. However, unmapped terms were associated with areas that can be uniquely common in radiology reporting. Likewise, there was a high percentage of lexical similarity between the terms used in the reports and the CHV-preferred terms. This has resulted in merely 27.3% of the sample terms to be considered lexically different after replacing them with their CHV counterparts.

### Content Coverage

A substantial amount of the effort that has been invested in the CHV involved broadening content coverage. The findings of this study suggest that the content coverage is perhaps not the highest priority improvement needed at this juncture. Nevertheless, the association between uncovered concepts and terms uniquely used in radiology reporting is notable. This is not surprising as the CHV concepts were created by finding terms that are frequently used in MedlinePlus [22], and many of the missing terms are less frequent and can even be absent in other medical texts. That, nevertheless, does not eradicate the importance of explaining such terms when they appear in patients’ radiology reports.

Another gap in coverage is abbreviations. Acronyms have a high risk of being ambiguous, which makes covering them difficult as their meanings can differ from one field to another. For example, PET and CAT in common terms can refer to types of animals. In contrast, it refers, in radiology, to positron emission tomography and computed axial tomography, respectively. This obstacle, however, can be solved in the implementation. MetaMap, for instance, can circumvent acronyms’ ambiguity by allowing users to predefine them before the mapping process.

### Lexical Similarity

Although a decent percentage of similarity had been expected, the result surpassed our expectations. It shows that about one of every 4 terms of interest is expressed differently in the CHV in comparison with radiology reports. This high level of similarity can be due to many reasons. It is either because some words in the reports are easy to grasp, a simpler explanation does not exist, or they are not explained to the consumer level in the CHV. All these factors are most likely relevant, but this matter requires more research to identify which one is dominant.

The usefulness of the CHV implementation, however, is illustrated when looking at terms that are lexically different. Explanations, for example, are expected to be easier in many contexts for people without background knowledge in pathology or anatomy. It is worth noting that adding explanations can possibly be problematic depending on the way it is used and the context. We can see that if we replace the term “cortex” with its given definition in the phrase “renal cortex,” without adjusting the sentence. Such a change would affect the readability and the coherence of the text. Another type of replacement that can increase understandability is terms that were modified by adding an extra word (ie, “heart” in “heart fibrillation”). It is, however, important to watch for redundancy, such as when a similar word is adjacent to the replaced term in the original report.

As there is a large percentage of similarity, introducing changes in these terms can lead to little benefit while introducing a risk of avoidable syntactic errors. Therefore, a simplification tool should include a grammatical layer to maintain the parts of speech after terms’ replacements. In fact, such a component is vital for all replaced terms, not only the similar ones. Another straightforward method to reduce syntactic errors is excluding terms from replacement if their CHV-preferred terms are lexically similar. This can be done by stemming the terms and by removing morphological affixes using an NLP algorithm. As a result, a tool would be prohibited from replacing words with others that have the same stem.

### Other Observations

When restricting semantic types, the inclusiveness of CHV concepts was negatively affected, missing potential mappings between terms in the radiology reports and concepts in the CHV. Not specifying them, in contrast, increased mapping errors that were related to the senses of the words. These errors made the text more difficult to read. Moreover, syntactic errors can also affect readability in a negative way. Some of them appeared in the text because of missing a ripple effect of change in the sentence after a one-to-one lexical replacement. Although this might be an inherent characteristic of lexical simplification, applying simple grammatical changes as part of the process would help to mitigate the problem.

Spelling errors are always a possibility in free narrative writing, which makes them an intrinsic challenge in NLP. In this study, misspelling appeared to be affecting lexical simplification more than grammatical errors as MetaMap does not recognize misspelled terms. One way to overcome this dilemma is by using noisy channel algorithms, which are able to predict intended words within a margin of error [34]. Furthermore, any type of premapping spell checking that corrects or excludes misspelled words is helpful.

Overall, the settings of this study were oriented toward its research objectives, mainly finding the content coverage and the lexical similarity. There are, however, possible optimizations to build a result-oriented translator, ranging from available MetaMap options, such as excluding unwanted semantic types and prespecifying acronyms, to postprocessing measures. All these optimizations would be intended mainly to help in simplifying terms without affecting the coherence of the text, which can be the major principle to create a successful lexical simplification process.

The CHV representation in the UMLS, specifically in the MRCONSO table, is an essential part of the translation methodology implemented in this work. It, however, has undermined finding the intended preferred terms for some of the concepts as two preferred terms can exist for one CUI, which does not happen when searching the CHV Web portal for the same CUIs. This may be because when CHV concepts were added to the UMLS, in some cases, two CHV concepts were found to fit one concept in the UMLS. Therefore, a change in the methodology applied in this work can be essential to create a successful lexical simplification tool based on the CHV.

Oh et al discuss a prototype project called “PORTER” that aimed to create patient-oriented reports by first establishing a “lay-language glossary” for MRI knee exams [35]. The CHV can be a good resource to start broadening such a project. Doing so would require a method for extracting preferred terms that can be useful for consumers in the specific matter. It is also possible that focusing the simplification process on the action items of the report would facilitate covering more types of medical imaging procedures while maintaining most of the benefit for the consumers. An actionable representation for patients in radiology can be restricted to the impressions section. Nevertheless, providing the rest of the report is still important for the purpose of transparency.

Generally, research in translation is an ongoing and a promising process. Google, for example, has announced in 2016 a new approach in machine translation for Google Translator using neural networks models for creating a better human-like translation between English and Chinese [36]. This emphasizes the importance of improving the CHV to support the advancements in translations when creating consumer-focused biomedical applications. In that process, automated methods to improve ontologies, known as Ontology Learning, can be advantageous to better encompass CHV [37]. A study investigating the “folksonomy” of terms published by patients in the platform “PatientsLikeMe” elucidates that about half of the terms did not have a match or a synonym in the UMLS [38]. Some of these terms might not have a direct concept that corresponds to them in the UMLS, but there might be a parent concept in the hierarchy where semantically broader concepts can be found. Keselman et al provides a framework of initiating “lay” tagged concepts that can be used for only consumer-based projects and still be linked to the UMLS hierarchy [39]. Doing so will help ontologies to cover more of CHV, which can help to bridge the gap between the two different ways consumers and clinicians describe medical phenomena.

### Limitations

The results of the study are based on a sample of 31 reports from a variety of radiology specialties. Although the reports include hundreds of terms used in the field, the results cannot be generalized to cover the vast realm of medical imaging. It, however, provides an overview that can pave the way for more targeted studies.

Although medical terms could be easy to define, general terms that describe medical phenomena are often vague. In some cases, choosing general terms to be included in the study might have depended on personal judgment despite following the inclusion criteria. Included words, nevertheless, had been defined before investigating what categorization they belong to (ie, missing) to eliminate the risk of bias.

This work measures the lexical but not the semantic similarity. Semantic similarity, also referred to as conceptual similarity, compares the conceptual meanings of words without considering their lexical similarity [40]. It is measured by mapping terms to an ontology and measuring their distance apart in a hierarchy. Semantic similarity can be investigated for many purposes. One main usage is to find similar documents despite different wording, which is very useful in information retrieval. Semantic similarity can be applied in the context of this study to investigate the accuracy of the translation in maintaining the conceptual meanings in the original reports. This measure does not only rely on the CHV word choices, but also on the mapping process, which serves a different but important purpose that complements what is intended in this study.

### Conclusions

The CHV provided an overall high content coverage of 88.5% for the terms found in the sample radiology reports. Yet, uncovered concepts are associated with areas that are uniquely common and important in radiology reporting, such as anatomical descriptions and radiation projections. The study also shows a high level of lexical similarity between CHV-preferred terms and original terms in the sample radiology reports, which have a plethora of medical jargon. This indicates that some CHV-preferred terms can be above the level of consumers’ comprehension. Such terms would require further simplification before successfully integrating the CHV into radiology-related applications that target consumers. Overall, our implementation shows that lexical simplification is not sufficient to simplify the reports for consumers, yet it can play an important role if used to complement other methods of simplification and explanation.

## Appendix

Multimedia Appendix 1 The full data of the terms' evaluation process.

## Abbreviations

CAT: computed axial tomography
CHV: consumer health vocabulary
CUI: concept unique identifier
HITECH: Health Information Technology for Economic and Clinical Health
LOINC: logical observation identifiers names and codes
MMI: MetaMap indexing
MRCONSO: concept names and sources table
MRI: magnetic resonance imaging
NLP: natural language processing
PET: positron emission tomography
UMLS: unified medical language system

## References

[1] Koh HK, Brach C, Harris LM, Parchman ML (2013). A proposed 'health literate care model' would constitute a systems approach to improving patients' engagement in care. Health Aff (Millwood).

[2] Xierali IM, Hsiao C, Puffer JC, Green LA, Rinaldo JCB, Bazemore AW, Burke MT, Phillips RL (2013). The rise of electronic health record adoption among family physicians. Ann Fam Med.

[3] (2009). Congress.

[4] Keselman A, Logan R, Smith CA, Leroy G, Zeng-Treitler Q (2008). Developing informatics tools and strategies for consumer-centered health communication. J Am Med Inform Assoc.

[5] Bruno MA, Petscavage-Thomas JM, Mohr MJ, Bell SK, Brown SD (2014). The “open letter”: radiologists' reports in the era of patient web portals. J Am Coll Radiol.

[6] Delbanco T, Walker J, Bell SK, Darer JD, Elmore JG, Farag N, Feldman HJ, Mejilla R, Ngo L, Ralston JD, Ross SE, Trivedi N, Vodicka E, Leveille SG (2012). Inviting patients to read their doctors' notes: a quasi-experimental study and a look ahead. Ann Intern Med.

[7] Delbanco T, Walker J, Darer JD, Elmore JG, Feldman HJ, Leveille SG, Ralston JD, Ross SE, Vodicka E, Weber VD (2010). Open notes: doctors and patients signing on. Ann Intern Med.

[8] Henshaw D, Okawa G, Ching K, Garrido T, Qian H, Tsai J (2015). Access to radiology reports via an online patient portal: experiences of referring physicians and patients. J Am Coll Radiol.

[9] Nielsen-Bohlman L, Panzer A, Kindig D (2004). Executive Summary. Health literacy: a prescription to end confusion.

[10] Keselman A, Slaughter L, Smith CA, Kim H, Divita G, Browne A, Tsai C, Zeng-Treitler Q (2007). Towards consumer-friendly PHRs: patients' experience with reviewing their health records. AMIA Annu Symp Proc.

[11] Siddharthan A (2006). Syntactic simplification and text cohesion. Res Lang Comput.

[12] Kandula S, Curtis D, Zeng-Treitler Q (2010). A semantic and syntactic text simplification tool for health content. AMIA Annu Symp Proc.

[13] Carroll J, Minnen G, Canning Y, Devlin S, Tait J (1998). Practical simplification of English newspaper text to assist aphasic readers. Proceedings of the AAAI-98 Workshop on Integrating Artificial Intelligence and Assistive Technology.

[14] Specia L, Jauhar S, Mihalcea R (2012). SemEval-2012 task 1: English lexical simplification.

[15] Crossley SA, Louwerse MM, McCarthy PM, McNamara DS (2007). A linguistic analysis of simplified and authentic texts. Modern Language J.

[16] McLaughlin GH (1969). SMOG grading-a new readability formula. J Read.

[17] Liu S, Moore R, Ganesan V, Nelson S, Wei Ma (2005). RxNorm: prescription for electronic drug information exchange. IT Prof.

[18] McDonald CJ, Huff SM, Suico JG, Hill G, Leavelle D, Aller R, Forrey A, Mercer K, DeMoor G, Hook J, Williams W, Case J, Maloney P (2003). LOINC, a universal standard for identifying laboratory observations: a 5-year update. Clin Chem.

[19] Aronson AR (2001). Effective mapping of biomedical text to the UMLS Metathesaurus: the MetaMap program. Proc AMIA Symp.

[20] Zeng QT, Tse T, Divita G, Keselman A, Crowell J, Browne AC, Goryachev S, Ngo L (2007). Term identification methods for consumer health vocabulary development. J Med Internet Res.

[21] Zielstorff RD (2003). Controlled vocabularies for consumer health. J Biomed Inform.

[22] Zeng QT, Tse T, Crowell J, Divita G, Roth L, Browne AC (2005). Identifying consumer-friendly display (CFD) names for health concepts. AMIA Annu Symp Proc.

[23] Zeng-Treitler Q, Goryachev S, Kim H, Keselman A, Rosendale D (2007). Making texts in electronic health records comprehensible to consumers: a prototype translator. AMIA Annu Symp Proc.

[24] Interactive MetaMap.

[25] SEER training.

[26] NationalRad.

[27] mtsamples.

[28] Radisphereradiology.

[29] (2016). NLM.

[30] Aronson A (2001). MetaMap evaluation. NLM.

[31] Van Rossum G (2007). Python programming language. Proceedings of the USENIX Annual Technical Conference.

[32] (2010). MySQL.

[33] Agrawal A, Elhanan G (2014). Contrasting lexical similarity and formal definitions in SNOMED CT: consistency and implications. J Biomed Inform.

[34] Brill E, Moore R (2000). An improved error model for noisy channel spelling correction.

[35] Oh SC, Cook TS, Kahn CE (2016). PORTER: a prototype system for patient-oriented radiology reporting. J Digit Imaging.

[36] Wu Y, Schuster M, Chen Z, Le Q, Norouzi M, Macherey W (2016). Google's neural machine translation system: bridging the gap between human and machine translation. arXiv.

[37] Maedche A (2002). Ontology Learning for the Semantic Web.

[38] Smith C, Wicks P (2008). PatientsLikeMe: consumer health vocabulary as a folksonomy. AMIA Annu Symp Proc.

[39] Keselman A, Smith CA, Divita G, Kim H, Browne AC, Leroy G, Zeng-Treitler Q (2008). Consumer health concepts that do not map to the UMLS: where do they fit?. J Am Med Inform Assoc.

[40] Pesquita C, Faria D, Falcão AO, Lord P, Couto FM (2009). Semantic similarity in biomedical ontologies. PLoS Comput Biol.

